# Cloning and characterization of the Type I Baeyer–Villiger monooxygenase from *Leptospira biflexa*

**DOI:** 10.1186/s13568-017-0390-5

**Published:** 2017-04-27

**Authors:** Romina D. Ceccoli, Dario A. Bianchi, Michael J. Fink, Marko D. Mihovilovic, Daniela V. Rial

**Affiliations:** 1 Área Biología Molecular, Departamento de Ciencias Biológicas, Facultad de Ciencias Bioquímicas y Farmacéuticas, Universidad Nacional de Rosario, CONICET, Suipacha 531, S2002LRK Rosario, Argentina; 2 Instituto de Química Rosario (IQUIR, CONICET-UNR), Facultad de Ciencias Bioquímicas y Farmacéuticas, Universidad Nacional de Rosario, Suipacha 531, S2002LRK Rosario Argentina; 3 Institute of Applied Synthetic Chemistry, Vienna University of Technology, Getreidemarkt 9/163, 1060 Vienna, Austria; 4 Department of Chemistry and Chemical Biology, Harvard University, Cambridge, MA USA

**Keywords:** Baeyer–Villiger monooxygenase, Biocatalysis, Leptospira, Ketones

## Abstract

Baeyer–Villiger monooxygenases are recognized by their ability and high selectivity as oxidative biocatalysts for the generation of esters or lactones using ketones as starting materials. These enzymes represent valuable tools for biooxidative syntheses since they can catalyze reactions that otherwise involve strong oxidative reagents. In this work, we present a novel enzyme, the Type I Baeyer–Villiger monooxygenase from *Leptospira biflexa*. This protein is phylogenetically distant from other well-characterized BVMOs. In order to study this new enzyme, we cloned its gene, expressed it in *Escherichia coli* and characterized the substrate scope of the Baeyer–Villiger monooxygenase from *L. biflexa* as a whole-cell biocatalyst. For this purpose, we performed the screening of a collection of ketones with variable structures and sizes, namely acyclic ketones, aromatic ketones, cyclic ketones, and fused ketones. As a result, we observed that this biocatalyst readily oxidized linear- and branched- medium-chain ketones, alkyl levulinates and linear ketones with aromatic substituents with excellent regioselectivity. In addition, this enzyme catalyzed the oxidation of 2-substituted cycloketone derivatives but showed an unusual selection against substituents in positions 3 or 4 of the ring.

**Electronic supplementary material:**

The online version of this article (doi:10.1186/s13568-017-0390-5) contains supplementary material, which is available to authorized users.

## Introduction

Baeyer–Villiger monooxygenases (BVMOs) comprise a rapidly growing group of enzymes recognized by their ability as oxidative biocatalysts. They are known to catalyze the oxidation of ketones to esters or lactones mainly, although other oxidation reactions have also been described for these enzymes such as epoxidations on carbon–carbon double bonds, sulfoxidations, and oxidations of boron and selenium compounds. The BVMOs are classified as Type I, Type II and atypical BVMOs. Type I BVMOs contain non-covalently bound flavin adenine dinucleotide (FAD) as coenzyme and strictly depend on reduced nicotinamide adenine dinucleotide phosphate (NADPH) as source of reducing power. They are the best studied BVMOs and the ones proven in scale-up biotransformation assays, as reviewed in (Balke et al. 2012; Bučko et al. 2016; Ceccoli et al. 2014; de Gonzalo et al. 2010; Leisch et al. 2011). Discovery and analysis of new BVMOs have been favored by intensive cloning strategies and high throughput methods that facilitated the expansion of the number of recombinantly available Type I BVMOs in the last decade (Torres Pazmiño et al. 2010). Several examples exist regarding the practical use of BVMOs as whole-cell biocatalysts in organic synthesis. Mihovilovic et al. (2006) reported the formal total synthesis of showdomycin, *trans*-kumausyne and goniofufurone analogs by chemoenzymatic approaches that included a key BVMO-mediated ketone oxidation to an intermediate chiral lactone. Few years later and by using a similar approach, Bianchi et al. (2013) reported the synthesis of (+) and (−) non-natural carba-*C*-nucleosides by accessing antipodal intermediate lactones in very good yields and high enantiomeric excess (*ee*). Recently, further applications of these antipodal lactones obtained by biooxidation mediated by two different BVMOs were reported (Rudroff et al. 2016). Moreover, BVMOs are also capable for combination within chemo-enzymatic transformations employing heterogeneous catalysis (Fink et al. 2013) as well as construction of artificial metabolic mini-pathways in vivo (Oberleitner et al. 2013) and in vitro (Oberleitner et al. 2014). Recently, demonstrations of prospective technological applications were presented: (1) BVMOs were utilized for the production of hydroxypropionic acid derivatives for bio-based bulk-chemical supply (Fink and Mihovilovic 2015), (2) an enzyme cascade allowed the synthesis of oligo-*ε*-caprolactone at more than 20 g/L when starting from 200 mM cyclohexanol by the sequential action of an alcohol dehydrogenase, a BVMO, and a subsequent direct ring-opening oligomerization of the ε-caprolactone formed using lipase A from *Candida antarctica* (Schmidt et al. 2015) and, (3) an in vivo biocatalytic cascade demonstrated the valorization of orange peel waste as starting material towards chiral carvolactone by a direct multi-step conversion that involved an oxygenation reaction catalyzed by a BVMO (Oberleitner et al. 2017).

*Leptospira biflexa* is a free-living bacterium of the genus *Leptospira*, order *Spirochaetales,* present in aquatic and soil environments. The genome of this saprophytic species had been sequenced in 2008 (Picardeau et al. 2008). As part of a bioinformatic survey for BVMOs sequences we decided to investigate the presence of genes coding for putative Type I BVMOs in the genome of *L. biflexa*. In this work, we describe the cloning of the gene coding for the Type I BVMO from *L. biflexa*, its expression in *Escherichia coli* and a complete characterization of this new BVMO (BVMO_Lepto_) as a whole-cell biocatalyst. The results are discussed and compared with data available in the literature for other Baeyer–Villiger biooxidations.

## Materials and methods

### Sequence alignment and phylogenetic analysis

Protein sequences of BVMOs (Additional file 1: Table S1) were aligned with MAFFT (Multiple Alignment using Fast Fourier Transform) version 7 (Katoh and Standley 2013). Phylogenetic trees were generated using the LG substitution model in PhyML 3.0 (Guindon et al. 2010). Branch support was calculated using the approximate likelihood ratio test (aLRT) with a Shimodaira-Hasegawa-like (SH-like) procedure. Phylogenetic trees were visualized using FigTree v1.3.1 (Rambaut and Drummond 2010).

### General

Chemical reagents as well as reagents for Molecular Biology were from commercial sources (Promega Corp., Madison, WI, USA; Invitrogen Corp., Carlsbad, CA, USA; Sigma-Aldrich Corp., St. Louis, MO, USA; Merck KGaA, Darmstadt, Germany; Genbiotech S.R.L., CABA, Argentina; BD (Becton, Dickinson and Company), Franklin Lakes, NJ, USA; Cicarelli Laboratorios, San Lorenzo, Argentina; Bio Basic Inc., Markham, ON, Canada; MP Biomedicals, Santa Ana, CA, USA). Substrates used in this study were either commercial or synthesized in our laboratories. Solvents were distilled before use.

### Plasmid construction, microbial strains and culture media

A DNA fragment containing the selected BVMO gene from *L. biflexa* (CP000786.1, Protein ABZ97795.1; previously YP_001839071.1) was obtained by polymerase chain reaction (PCR) of genomic DNA using primers 5′-GATTCGCTAGCATGACAACATCAGGTTTTAG-3′ and 5′-ACTGCCTCGAGTTATTGGGTGGTGAGAC-3′ that contain *Nhe*I and *Xho*I recognition sites, respectively. The PCR amplification was performed with *Pfu* DNA polymerase (Promega Corp, Madison, WI, USA) according to the manufacturer protocol and supplemented with 5% (v/v) dimethyl sulfoxide. The amplified DNA fragment corresponding to the predicted length (1489 base pairs) was digested and ligated into compatible sites of pET-TEV plasmid (Houben et al. 2007) to produce the pHLb01 plasmid. All DNA purifications were carried out using the Wizard^®^ SV Gel and PCR Clean-Up System (Promega Corp, Madison, WI, USA). The recombinant plasmid was isolated using Wizard^®^ Plus SV Miniprep DNA Purification System (Promega Corp, Madison, WI, USA) and its sequence was confirmed by DNA sequencing. *E. coli* strains were chemically transformed with the plasmid by standard procedures (Sambrook et al. 1989), and grown at 37 °C in LB-agar medium (5 g/L yeast extract, 10 g/L peptone, and 5 g/L NaCl, 15 g/L agar) supplemented with 50 μg/mL kanamycin.

The genomic DNA from *L. biflexa* serovar Patoc strain Patoc 1 (Paris) was kindly provided by Prof. Eduardo A. Ceccarelli from Instituto de Biología Molecular y Celular de Rosario, Rosario, Argentina and Prof. Mathieu Picardeau from Institut Pasteur, Paris, France (Picardeau et al. 2008). The strain *L. biflexa* serovar Patoc strain Patoc1 (Paris) (CRBIP6.1176) is maintained in the Centre de Ressources Biologiques de l’Institut Pasteur, Paris, France.

### Protein expression

A pre-culture of *E. coli* BL21(DE3) cells transformed with pHLb01 plasmid was grown overnight in LB medium supplemented with 50 μg/mL kanamycin. Then, fresh LB medium with kanamycin was inoculated with the overnight pre-culture [2% (v/v)] and incubated at 37 °C until optical density OD_600_ = 0.4–0.6 was reached. Next, isopropyl β-d-1-thiogalactopyranoside (IPTG) was added to induce recombinant gene expression at 0.3 mM final concentration and the culture was transferred to 24 °C. In order to analyze flavoprotein production, the cells were harvested by centrifugation after overnight induction and resuspended in 50 mM Tris–HCl buffer, pH 8 containing 150 mM NaCl, 0.05 mg/mL lysozyme, 0.1 mM benzamidine and 0.5% (v/v) triton X-100. The cell homogenate was centrifuged at 4 °C for 15 min at 12,000*g*. The presence of BVMO_Lepto_ in the soluble and insoluble fractions was analyzed by SDS-PAGE on 12% polyacrylamide gel.

### Whole-cell biotransformations

Biotransformations of linear- and branched-chain ketones as well as some aromatic and cyclic ketones were carried out in 10 mL cultures. Recombinant gene expression was induced at 0.3 mM IPTG and substrates were added as solutions prepared in ethanol (0.5 or 0.1 mg/mL final concentration, as required). After 24 h of reaction at 24 °C, analytical samples were centrifuged and the supernatant (0.7 mL) was extracted with diethyl ether (0.7 mL) supplemented with 0.5 mg/mL 1,3,5-trimethylbenzene as internal standard. The organic phase was removed, dried with anhydrous Na_2_SO_4_, and analyzed by gas chromatography-mass spectrometry (GC–MS; Shimadzu GCMS-QP2010 Plus from Shimadzu Corporation, Kyoto, Japan) using an achiral and/or a chiral capillary column (SPB-1 Capillary GC Column or β-DEX 325, respectively, 30 m × 0.25 mm ID, 0.25 μm film, from Supelco, Bellefonte, PA, USA). Reaction products were predicted by GC–MS or by comparison with reference biotransformations, when possible. Other ketones including levulinic esters and most of cyclic ketones were assayed using 24-well plates following the general procedure described in (Rial et al. 2008a, b) with minor modifications. In this case, β-cyclodextrin (4.0 mM) was supplemented as a cell membrane transfer agent; the culture was thoroughly shaken and then divided into 1.0 mL aliquots. Substrates were added as solutions in 1,4-dioxane (4.0 mM final concentration), the plates were sealed with adhesive film and incubated at 24 °C for 24 h. After centrifugal separation of the cell mass, samples were prepared by extraction of the biotransformation culture (0.5 mL) with ethyl acetate (1.0 mL) containing 1.0 mM methyl benzoate as internal standard and analyzed by chiral-phase GC-FID (Thermo Scientific Trace or Focus, Thermo Fisher Scientific, Waltham, MA, USA) using columns BGB175 or BGB173 (30 m × 0.25 mm ID, 0.25 μm film) from BGB Analytik AG (Boeckten Switzerland) or achiral-phase GC-FID (Thermo Scientific Trace or Focus) using column TR5-MS (15 m × 0.25 mM ID, 0.10 µm film) from Thermo Fisher Scientific, Waltham, MA, USA. All biotransformations were performed as triplicates; conversion and selectivity are reported. Whenever possible, optical rotation signs of products were assigned on the basis of published reference biotransformations.

## Results

### Protein sequence analysis

**Fig. 1:**
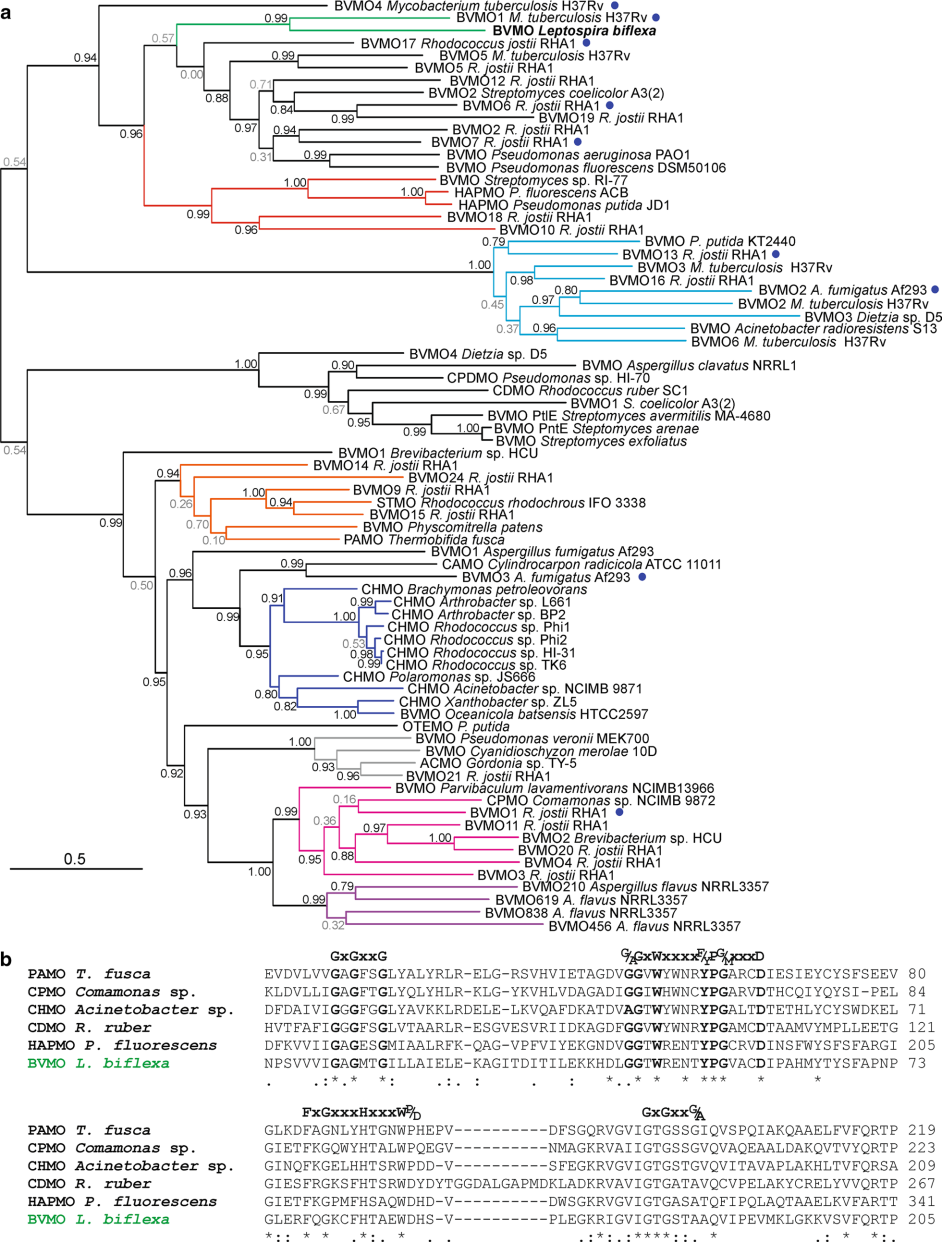
Sequence homology analysis of BVMO_Lepto_. **a** Maximum-likelihood phylogenetic tree of recombinant BVMOs. The *scale bars* indicate the number of substitutions per site per unit of branch length. The aLRT values are shown at the nodes: >0.75 (*black*) and <0.75 (*grey*). The *colors of branches* indicate the groups I (*pink*), II (*orange*), III (*blue*), IV (*red*), V (*cyan*), VI (*violet*) and VII (*gray*) as previously defined (Ferroni et al. 2014; Szolkowy et al. 2009). *Blue circles* indicate recombinant BVMOs that were insoluble or their activity was not detected. BVMO from *L. biflexa* is indicated in *bold*. Protein sequences with their corresponding accession numbers are listed in Additional file 1: Table S1. **b** Multiple sequence alignment of six representative BVMOs belonging to different clades. The partial alignment of PAMO from *T. fusca* (Q47PU3), CHMO from *Acinetobacter* sp. NCIMB 9871 (BAA86293), HAPMO from *P. fluorescens* ACB (AAK54073), CPMO from *Comamonas* sp. NCIMB 9872 (BAC22652), CDMO from *R. ruber* SC1 (AAL14233) and BVMO from *L. biflexa* (ABZ97795) is shown. The two Rossmann-fold motifs (GxGxxG/A) and the two consensus sequences of Type I BVMOs (G/AGxWxxxxF/YPG/MxxxD and FxGxxxHxxxWP/D) are written in *bold*

We investigated the presence of BVMOs in *L. biflexa* by bioinformatic approaches and detected only one sequence corresponding to a putative Type I BVMO. In order to study this protein sequence, phylogenetic relationships were established amongst recombinantly available BVMOs both from eukaryotes and prokaryotes (Fig. 1; Additional file 1: Table S1). The topology of the un-rooted tree (Fig. 1a) showed different clades of Type I BVMO sequences corresponding to the previously defined groups I to VII (Ferroni et al. 2014; Szolkowy et al. 2009). The BVMO_Lepto_ shares 29–33% sequence identity with proteins of the group IV that includes BVMOs with an N-terminal extension such as BVMO18 from *Rhodococcus jostii* RHA1 (30% sequence identity) and HAPMO from *Pseudomonas putida* JD1 (33% sequence identity) (Additional file 1: Table S1). The sequence of the putative BVMO_Lepto_ clusters with several divergent Type I BVMOs with variable and/or partially characterized substrate scope and shares approximately 34–44% sequence identity with BVMOs in this clade. Recombinant expression of several BVMOs belonging to this cluster was negligible or its activity was not detected: BVMO6, BVMO7 and BVMO17 from *R jostii* (Riebel et al. 2012; Szolkowy et al. 2009), and BVMO1 from *Mycobacterium tuberculosis* H37Rv (Bonsor et al. 2006) (Fig. 1a). According to our analysis, the BVMO_Lepto_ groups in a subclade together with the BVMO1 from *M. tuberculosis* H37Rv (Fig. 1a) and they share 44% sequence identity (Additional file 1: Table S1). Taking all this into account, the BVMO_Lepto_ is an interesting candidate to be functionally produced in *E. coli* and characterized as a biocatalyst in order to contribute to the knowledge of Type I BVMOs.

The two Rossmann-fold motifs for dinucleotide-binding (GxGxxG/A) and the characteristic Type I BVMO consensus sequences (G/AGxWxxxxF/YPG/MxxxD and FxGxxxHxxxWP/D) are conserved in the new BVMO_Lepto_, as it is shown in a multiple sequence alignment of BVMO_Lepto_ and representative Type I BVMOs from different clades (Fig. 1b; Additional file 1: Figure S1): 4-hydroxyacetophenone monooxygenase (HAPMO) from *Pseudomonas fluorescens* ACB (32% sequence identity, group IV), cyclohexanone monooxygenase (CHMO) from *Acinetobacter* sp. NCIMB 9871 (30% sequence identity, group III), phenylacetonone monooxygenase (PAMO) from *Thermobifida fusca* (27% sequence identity, group II), cyclododecanone monooxygenase (CDMO) from *Rhodococcus ruber* SC1 (28% sequence identity, group VII) and cyclopentanone monooxygenase (CPMO) from *Comamonas* sp. NCIMB 9872 (27% sequence identity, group I). The BVMO_Lepto_ consists of 488 amino acids and it is characterized by the lack of 52 residues that comprise one helical turn (positions 257–259), one helix (positions 262–276) and a second short helix (positions 278–284) in the polypeptide chain of the CHMO from *Rhodococcus* sp. HI-31 (Additional file 1: Figure S2).

### Cloning and expression of BVMO_Lepto_ in *E. coli*

**Fig. 2:**
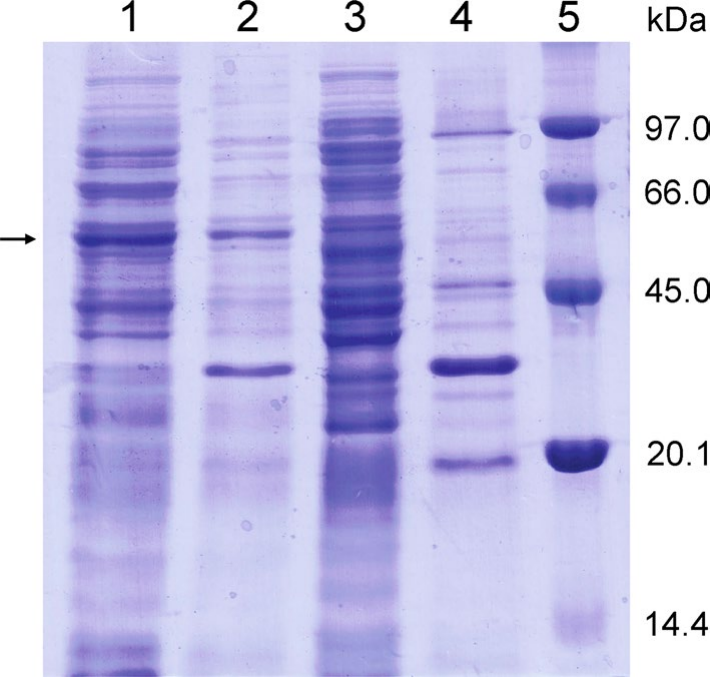
SDS-PAGE of recombinant BVMO_Lepto_. Samples corresponding to the soluble (*lane 1*) and insoluble (*lane 2*) fractions of protein extracts of *E. coli* BL21(DE3)/pHLb01 and the soluble (*lane 3*) and insoluble (*lane 4*) fractions of BL21(DE3) protein extract were subjected to 12% SDS-PAGE followed by Coomassie Blue staining. *Lane 5* molecular marker. The *arrow* indicates the protein band corresponding to the recombinant BVMO_Lepto_

The gene coding for BVMO_Lepto_ was amplified by PCR from genomic DNA and cloned into the pET-TEV vector as it is described in “Materials and methods”. Upon induction of gene expression, the full-length BVMO_Lepto_ was produced as fusion to an N-terminal His-Tag (expected size 54 kDa). The presence of BVMO_Lepto_ in protein extracts of induced *E. coli* cells was analyzed by SDS-PAGE and Coomassie Blue staining (Fig. 2). A major band of estimated 54 kDa that matched with the expected size of the recombinant BVMO_Lepto_ was detected both in the soluble and insoluble fractions of the induced culture. Approximately 60% of the protein was obtained in soluble form (Fig. 2, lanes 1 and 2). This band was not detected in *E. coli* BL21(DE3) cells, used as controls (Fig. 2, lanes 3 and 4). In consequence under the experimental conditions tested, recombinant gene expression yielded the BVMO_Lepto_ protein for biotransformations in whole-cell systems.

### Assessment of the substrate profile of BVMO_Lepto_ as a whole-cell biocatalyst

**Fig. 3:**
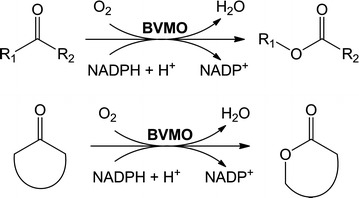
General Baeyer–Villiger oxidation catalyzed by BVMOs

In order to evaluate the potential of BVMO_Lepto_ as a biocatalyst, we decided to perform whole-cell biotransformation assays. This approach has the advantage of providing NADPH in vivo during the course of reaction. In this study, we aimed at detecting Baeyer–Villiger oxygenation of ketones (Fig. 3). Whole-cells expressing the BVMO_Lepto_ were challenged with a collection of ketones with diverse structural characteristics, namely acyclic, cyclic, aromatic, and fused ketones.

In order to explore the ability of this BVMO to oxidize linear- and branched-chain ketones, a set of aliphatic ketones and three alkyl levulinates were tested as substrates. Linear- and branched-chain ketones (**1**–**6**) were oxidized with very good conversion and excellent regioselectivity towards the normal ester product formed by the insertion of the oxygen atom on the side of the longer or branched alkyl chain (Table 1). However, cellular lysis or growth inhibition was observed when biotransformations were carried out with the 2-methyloctan-4-one (**7**) and with nonan-2-one (**8**) and its derivative **9** under standard conditions. In order to overcome this drawback, we decided to lower the concentration of ketones **7**, **8** and **9** to 0.1 mg/mL. Consequently, excellent conversions of **8** and **9** were achieved whereas moderate conversion of ketone **7** was detected even at low substrate concentration (Table 1).

**Table 1:** Biotransformations of linear- and branched-chain ketones mediated by BVMO_Lepto_

No	Substrate	Product	Conv (%)^a^
**1**			65
**2**			>99
**3**			82
**4**			>99
**5**			>99
**6**			98
**7**			88^b^
**8**			>99^b^
**9**			>99^b^
**10**			>99
**11**			>99
**12**			>99

^a^Relative conversion (Conv) of starting material to ester as determined by GC-FID or GC–MS

^b^Relative conversion of substrate to ester measured using 0.1 mg/mL of starting material

The esters shown in Table 1, entries 1-9 (i.e. *tert*-butyl acetate, propyl acetate, ethyl propionate, isobutyl acetate, butyl propionate, 4-methylpent-3-en-1-yl acetate, heptyl acetate, pentyl butyrate) are important aroma compounds, pesticides or solvents of great interest for the industry. Conventional procedures for the synthesis of these esters involve the use of chemical catalysts, organic solvents, high temperatures and/or refluxing conditions. In contrast, the biooxidation approach towards them is an advantageous alternative to the traditional organic procedures, as high conversions can be achieved by a simple, clean, and efficient method. To the best of our knowledge, this work is the first report of a Baeyer–Villiger biooxidation of ketones **1**, **3**–**7** and **9** to afford their valuable normal esters. Alkyl levulinates **10**, **11** and **12** were readily accepted and oxidized by the BVMO_Lepto_ biocatalyst with excellent conversion (>99%) and selectivity, producing only substituted propionates (Table 1). These compounds can be easily hydrolyzed to 3-hydroxypropionic acid, which is considered as a value-added chemical precursor or building block for several bulk chemicals.

Whole-cells expressing BVMO_Lepto_ showed a trend for the oxidation of the aromatic ketones (Table 2). Acetophenone (1-phenylethanone, **13**) was not a substrate for the biocatalyst but phenylacetone (1-phenylpropan-2-one, 1**4**), 1-(*p*-tolyl)propan-2-one (**15**), 1-phenylbutan-2-one (**16**), 4-phenylbutan-2-one (**17**) and 4-(4-hydroxyphenyl)butan-2-one (**18**) were fully converted to the normal esters (>99%). The biooxidations on ketones **14**, **15**, and **16** give access to benzyl acetate, 4-methylbenzyl acetate or benzyl propionate, respectively, which are highly used in the fragrance, cosmetic and food industries as flavor and aroma compounds (McGinty et al. 2012; Surburg and Panten 2006). According to our information, neither 4-methylbenzyl acetate nor benzyl propionate has been obtained by BVMO-mediated biooxidations before.

**Table 2:** Biotransformation of aromatic ketones mediated by BVMO_Lepto_

No	Substrate	Product	Conv (%)^a^
**13**		–	nc
**14**			>99
**15**			>99
**16**			>99
**17**		nd	>99
**18**			>99

*nc* no conversion, *nd* not determined

^a^Relative conversion (Conv) of starting material to ester as determined by GC-FID or GC–MS

In order to examine the ring size effect of cyclic ketones and the influence of different substituents on the biocatalytic performance of BVMO_Lepto_, representative cyclobutanone, cyclopentanone and cyclohexanone derivatives were evaluated as substrates of BVMO_Lepto_. The biocatalyst preferred cyclohexanone derivatives with the substitution in position 2 over substituents in position 3 or 4 of the ring (Table 3, compounds **19**–**25**). Oxygen insertion and migration occurred at the more substituted carbon of compounds **19**–**22**, thus giving access to normal lactones exclusively in very good *ee* with an enantioselectivity trend similar to that observed for CHMO from *Acinetobacter* sp. NCIMB 9871 (Stewart et al. 1996, 1998). In addition, the set of cyclopentanone derivatives showed an intriguing behavior as well (Table 3). In this series, the 2-benzylcyclopentanone (**26**) and 3-(2-oxocyclopentyl)propanenitrile (**27**) were accepted and showed very good conversions but *trans*-3-methyl-2-pentylcyclopentanone (**28**), 3-methylcyclopent-2-enone (**29**), and 3-methylcyclopentanone (**30**) were not substrates for BVMO_Lepto_ in whole-cell biotransformations, in agreement with the preference observed for the 2-substituted cyclohexanone derivatives (Table 3). Similarly, the 3-substituted cyclobutanones that are oxidation-prone ketones, were poorly oxidized or not converted at all by BVMO_Lepto_ under the assayed conditions (Table 3, compounds **31**–**33**), showing that these ketones are not substrates for this BVMO.

**Table 3:** Biooxidation of substituted cyclic ketones by BVMO_Lepto_

No	Substrate	Conv (%)^a^	*ee*_P_ (%)^b^
**19**		55	68(−)
**20**		36	98(−)
**21**		97	nd
**22**		16	88(−)
**23**		nc	na
**24**		nc	na
**25**		nc	na
**26**		86	nd
**27**		98	nd
**28**		nc	na
**29**		nc	na
**30**		nc	na
**31**		9	21(+)
**32**		5	27(+)
**33**		nc	na

*nc* no conversion, *nd* not determined, *na* not applicable

^a^Relative conversion (Conv) of starting material to lactone as determined by GC

^b^Enantiomeric excess of the product (*ee*_P_) was determined by chiral phase GC. The sign of specific rotation is indicated in brackets and was assigned according to the literature for reference biotransformations

**Fig. 4:**
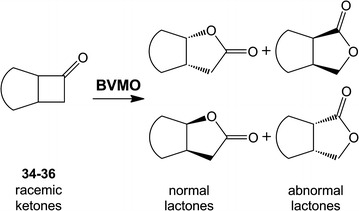
Baeyer–Villiger biooxidation of racemic fused ketones **34**, **35** and **36** to normal and abnormal lactones

The selectivity of the oxygenation of the fused cyclobutanones was analyzed by chiral phase GC. Figure 4 shows the four possible products of biooxidation on racemic fused cyclobutanones. The oxidation of the racemic *cis*-bicyclo[3.2.0]hept-2-en-6-one (**34**) by CHMO from *Acinetobacter* sp. NCIMB 9871 is a well-known biotransformation reaction that allows access to approximately 1:1 ratio of normal and abnormal lactones with high optical purities (Alphand and Furstoss 1992). Like the CHMO from *Acinetobacter* sp. NCIMB 9871, the BVMO_Lepto_ oxidized the fused cyclobutanone **34,** albeit with poorer *ee* of both normal (50%) and abnormal lactones (23%) (Table 4). Besides, the conversion and regioselectivity of BVMO_Lepto_ on ketones **35** and **36** were low but with an unusual trend towards the abnormal lactones. In both cases, abnormal lactones were produced in high optical purity (*ee* 95% and >99%, respectively).

**Table 4:** Biooxidation of fused cyclobutanones mediated by BVMO_Lepto_

No	Substrate	BVMO *Leptospira biflexa*		Reference biotransformation using CHMO *Acinetobacter* sp. NCIMB 9871 as biocatalyst		
		Conv^a^ (%)	*ee*_N_, *ee*_ABN_ (%)^b^	Yield (%)	*ee*_N_, *ee*_ABN_ (%)^b^	References
		N:ABN (%)		N:ABN (%)		
**34**		94	50(−), 23(−)	74	95(−), >99(−)	Mihovilovic et al. (2005b)
		50:50		51:49		
**35**		8	44(−), 95(−)	80	60(−), >95(−)	Alphand and Furstoss (1992)
		27:73		65:35		
**36**		47	96(nd), >99(nd)	84	44(nd), >99(nd)	Mihovilovic et al. (2008)
		19:81		70:30		

*N:ABN* ratio of normal:abnormal lactones, *nd* not determined

^a^Relative conversion (Conv) of starting material to lactone as determined by chiral phase GC

^b^Enantiomeric excess for normal lactone (*ee*_N_) and abnormal lactone (*ee*_ABN_). The sign of specific rotation is indicated in brackets and was assigned according to the literature for reference biotransformations

## Discussion

As part of a survey for putative Type I BVMOs sequences in public databases, we investigated the presence of BVMOs in *L. biflexa* by bioinformatic analysis and detected only one gene encoding a Type I BVMO in *L. biflexa*. In this work, we present the Type I BVMO from *L. biflexa*, its protein sequence analysis and phylogenetic studies. In addition, we report the cloning, heterologous expression in *E. coli* and biocatalyst assessment of this novel BVMO in whole-cell systems.

The phylogenetic analysis based on known Type I BVMOs indicates that our new BVMO_Lepto_ belongs to a clade that remains only partially explored for biocatalysis (Fig. 1a). Indeed, the BVMO_Lepto_ and the BVMO1 from *M. tuberculosis* H37Rv (Accession Number CAA97398) (Bonsor et al. 2006) group together in a subclade of the phylogenetic tree. The BVMO_Lepto_ is the first enzyme of this subclade to be functionally produced in *E. coli* and characterized as a Baeyer–Villiger monooxygenase since the gene coding for BVMO1 from *M. tuberculosis* cloned in plasmid pDB1 was expressed poorly if at all and no activity could be measured with ketones as substrates (Bonsor et al. 2006). The BVMO_Lepto_ consists of 488 amino acids and lacks 52 residues that comprise one helical turn and two helices in the polypeptide chain of the CHMO from *Rhodococcus* sp. HI-31 (Yachnin et al. 2014) as predicted by homology modeling (Additional file 1: Figures S1, S2). This is a feature that BVMO_Lepto_ shares with the others BVMOs of the same clade (Fig. 1a). In the crystallographic structure of the CHMO from *Rhodococcus* sp. HI-31, these helices are exposed to the solvent (Additional file 1: Figure S1). The typical consensus sequences of Type I BVMOs are well conserved in BVMO_Lepto_ (Fig. 1b).

The BVMO_Lepto_ coding sequence was cloned and overexpressed in *E. coli* yielding approximately 60% of soluble recombinant protein (Fig. 2). In order to investigate its substrate scope for possible synthetic applications of this BVMO and to compare its performance with other BVMOs, we have challenged BVMO_Lepto_ with a broad variety of ketones of different sizes and bearing diverse functional substituents.

Although some model aliphatic linear ketones have been evaluated as substrates for BVMOs (Beneventi et al. 2013; Bisagni et al. 2014a, b; Bonsor et al. 2006; Ferroni et al. 2014; Fraaije et al. 2004; Kirschner et al. 2007; Kotani et al. 2007; Rehdorf et al. 2007, 2009, 2010a, b; Riebel et al. 2012; Szolkowy et al. 2009; van Beek et al. 2014; Völker et al. 2008), in this work, we present a systematic analysis of BVMO_Lepto_-mediated biooxidations of a broad range of unsubstituted linear ketones, linear-branched ketones and linear ketones with aromatic substituents. To the best of our knowledge, about half of the ketones shown in Tables 1 and 2 have never been reported as BVMO substrates before.

Amongst the tested linear and linear-branched ketones, this biocatalyst showed a clear preference towards medium-length, linear- and branched-chain ketones as substrates, reaching full conversions in most cases under standard biotransformation experiments. These reactions proceeded with excellent selectivity as judged by the insertion of the oxygen atom on the side of the longer or branched alkyl chain, producing the so-called normal regioisomeric ester in each case only (Table 1). Only pentan-2-one and nonan-2-one out of the ketones listed in Table 1, had been evaluated as substrates for BVMOs in previous years (Bisagni et al. 2014b; Kotani et al. 2007; Rehdorf et al. 2007; Völker et al. 2008). So far, the biooxidation of penta-2-one have been measured following the consumption of NADPH at 340 nm in vitro (Kotani et al. 2007; Völker et al. 2008). Similarly to BVMO_Lepto_-mediated biotransformation, the BVMO3 from *Dietzia* sp. D5 (Bisagni et al. 2014b) and the BVMO from *P. putida* KT2440 (Rehdorf et al. 2007) oxidized nonan-2-one to heptyl acetate. On the other hand, Bisagni et al. (2014b) reported the formation of normal and abnormal esters of 3-keto substrates by the BVMO3 from *Dietzia* sp. D5. In BVMO_Lepto_-mediated biooxidations of ketones **5**, **7** and **9**, the keto group at the second, third or fourth C-atom did not affect regioselectivity since the corresponding normal esters were obtained in all cases with excellent conversions after 24 h of biotransformation (Table 1). When we explored the biotransformation of 2-methyloctan-4-one (**7**), nonan-2-one (**8**) and its derivative **9** under standard conditions, we detected cellular lysis or growth inhibition. A similar observation had been reported for the biooxidation of nonan-2-one by the BVMO from *P. putida* KT2440 under growing conditions but, in that case, the authors observed no cellular growth and no conversion of the ketone (Rehdorf et al. 2007). By reducing the concentration of ketones **7**, **8** and **9** in the biotransformation reactions, we were able to achieve high conversions (Table 1).

Levulinic esters are valuable precursors of the 3-hydroxypropionic acid, a versatile compound which may be employed as platform chemical for the synthesis of 1,3-propanediol, acrylamide, acrylic acid and methyl acrylate, among others. The 3-hydroxypropionic acid can be synthesized chemically and biologically as reviewed in (Kumar et al. 2013). Besides, the enzyme-mediated Baeyer–Villiger oxidation of levulinic esters followed by hydrolysis may enable a perspective for a practical and simple alternative to produce 3-hydroxypropionic acid. In this context, the biocatalytic Baeyer–Villiger oxidation of alkyl levulinates in water under ambient conditions was demonstrated recently (Fink and Mihovilovic 2015). In the same work, it was also shown the Baeyer–Villiger oxidation of ethyl levulinate on gram scale to afford ethyl 3-acetoxypropionate (Fink and Mihovilovic 2015). In that study, 13 different BVMOs were evaluated on three alkyl levulinates. The best results were obtained with CPMO from *Comamonas* sp. NCIMB 9872 and CDMO from *R. ruber* SC1 on butyl levulinate (~90–95% conversion), although their activity on methyl or ethyl levulinates was moderate (Fink and Mihovilovic 2015). In the present work, we observed that the BVMO_Lepto_, which is distantly related to CPMO from *Comamonas* sp. NCIMB 9872 and CDMO from *R. ruber* SC1, was able to accept and oxidize levulinic esters with excellent conversion (>99%) and selectivity (Table 1) allowing easy access to pure propionates derivatives, thus providing a very interesting alternative tool for these biotransformations.

In addition, all tested linear ketones with aromatic substituents, except acetophenone (**13**), were excellent substrates for BVMO_Lepto_ (Table 2). Acetophenone is not a substrate for CHMO from *Acinetobacter* sp. NCIMB 9871 either, although it has been accepted by three fungal BVMOs from *Aspergillus flavus* NRRL3357 (Ferroni et al. 2014) and by HAPMO from *P. fluorescens* ACB (Kamerbeek et al. 2003). This preference against acetophenone derivatives and for phenylacetone (**14**) is not novel, since the well-known PAMO from *T. fusca* showed the same behavior (Fraaije et al. 2005). To our knowledge, this is the first work that reports the synthesis of 4-methylbenzyl acetate or benzyl propionate by BVMO-mediated biotransformations of ketones **15** or **16**. In the series shown in Table 2, it is clear that BVMO_Lepto_ oxidizes linear aromatic ketones with insertion of the oxygen atom to the more substituted carbon. In the case of 1-phenylbutan-2-one (**16**), where both *α*-carbon atoms are equally substituted, the migratory aptitude of the *α*-carbon atoms seemed to be primarily determined by the higher nucleophilicity of the *α*-carbon bonded to the aromatic ring due to its donating electronic effect compared with the methyl group. The 4-phenylbutan-2-one (**17**) and the 4-(4-hydroxyphenyl)butan-2-one (**18**) have been evaluated as substrates on the four BVMOs from *A. flavus* NRRL3357 (Ferroni et al. 2014). The authors reported that longer distances between the carbonyl group and the aromatic ring, and a hydroxy substitution on the *para* position of the ring resulted in relative low conversions for some of the enzymes. However, these features did not affect the excellent conversions observed with BVMO_Lepto_ after 24 h, indicating that it may be the best biocatalyst for this kind of transformations reported to date.

In this work, we present biotransformation results after 24 h of reaction. Although kinetic resolution processes could be possible for racemic substrates, these studies might be the matter of future research. Amongst the series of cyclic ketones, it is worth noting that the oxidation of cyclohexanone derivatives was very selective towards structures with substituents in *α*-position to the carbonyl group, as evidenced by the lack of reaction on 3,5-dimethylcyclohexanone (**23**), 4-methylcyclohexanone (**24**) or 4-hydroxy-4-methylcyclohexanone (**25**) (Table 3). A similar substitution preference was observed for the oxidation of mono- and disubstituted cyclopentanones (**26**–**30**) (Table 3). The BVMO4 from *Dietzia* sp. D5 also revealed a decrease in activity in the series of 2-, 3- and 4-methylcylohexanones by spectrophotometric measurements (Bisagni et al. 2014a). However, this is not a general behavior since oxidations of 3- or 4-substituted cyclohexanones are well-established reactions for the typical cycloketones-converting BVMOs such as CHMO from *Acinetobacter* sp. NCIMB 9871 (Mihovilovic et al. 2001; Stewart et al. 1998; Taschner and Black 1988), CHMO from *Xanthobacter* sp. ZL5 (Rial et al. 2008a, b), CPMO from *Comamonas* sp. NCIMB 9872 (Iwaki et al. 2002), CDMO from *R. ruber* SC1 (Fink et al. 2012), CPDMO from *Pseudomonas* sp. strain HI-70 (Fink et al. 2011; Iwaki et al. 2006), and CHMO from *Polaromonas* sp. strain JS666 (Alexander et al. 2012). Therefore, the strict selection against substituents in position 3 and 4 of the ring seems to be a unique behavior of BVMO_Lepto_ since to the best of our knowledge, such clear selectivity has never been reported for another BVMO before. These intriguing results led us to consider conducting three-dimensional protein studies and molecular docking simulations in the future in order to investigate the steric hindrance that prevent interaction of these substrates with the enzyme and shed some light on the mode of their binding to the active site of BVMO_Lepto_. In line with these observations, the 3-substituted cyclobutanones tested (**31**–**33**) were very poor substrates or not substrates at all for BVMO_Lepto_ (Table 3). BVMOs that cluster together with BVMO_Lepto_ have never been evaluated on these ketones before, thus available data correspond to distant BVMOs. The HAPMO from *P. fluorescens* ACB is the phylogenetically closest BVMO that has been evaluated on ketones **31** and **32**; this enzyme showed modest yield and *ee* for the desymmetrization of **31** and **32** (Mihovilovic et al. 2005a). However, the CHMO-type enzymes, which belong to the phylogenetic group III, readily catalyze the desymmetrization of prochiral ketones **31**–**33** to the corresponding lactones with high *ee* (Alexander et al. 2012; Alphand et al. 1998; Rial et al. 2008a; Rudroff et al. 2007). The racemic *cis*-bicyclo[3.2.0]hept-2-en-6-one (**34**) is a well-known substrate for CHMO from *Acinetobacter* sp. NCIMB 9871 and for many other BVMOs (Alexander et al. 2012; Alphand and Furstoss 1992; Ferroni et al. 2014; Fink et al. 2011; Mihovilovic et al. 2005b, 2008; Rial et al. 2008b). After 24 h, the BVMO_Lepto_ oxidized this racemic substrate almost completely to equal amounts of normal and abnormal lactones (Fig. 4) but, unlike CHMO from *Acinetobacter* sp. NCIMB 9871, the *ee* of each regioisomeric lactone was poor (Table 4), The oxidation of the fused ketone **35** or **36** by BVMO_Lepto_ preferentially gave abnormal lactones. These compounds (**35** or **36**) have been reported as substrates on approximately 10 Type I BVMOs, and in all cases except the BVMO5 from *M. tuberculosis* H37Rv (Snajdrova et al. 2006), approximately equal amounts of regioisomeric lactones or normal lactones have been preferentially produced (Alphand and Furstoss 1992; Fink et al. 2012; Mihovilovic et al. 2005a, 2008; Rial et al. 2008b).

The substrate scope of the enzymes that locate to the clade of the phylogenetic tree together with BVMO_Lepto_ (Fig. 1a) is still unclear due to the little information available regarding these enzymes. Nevertheless, particular observations about the substrate profile of some of them are worth noting. The BVMO5 from *M. tuberculosis* H37Rv is the only previous report of a BVMO able to oxidize ketones **35** and **36** preferentially to the abnormal lactones (Snajdrova et al. 2006). Besides, the BVMO from *P. fluorescens* DSM 50106 showed a narrow substrate profile with high selectivity towards aliphatic open-chain ketones (C_8_, C_10_), but no cyclic or aromatic ketones were accepted (Kirschner et al. 2007). Other members, such as the previously mentioned BVMO5 from *M. tuberculosis* H37Rv or the BVMOs 2, 5 and 19 from *R. jostii* also accepted aliphatic open-chain ketones (Bonsor et al. 2006; Riebel et al. 2012). Despite the fact that the ability to oxidize linear ketones is not exclusively limited to enzymes of this clade, a certain preference for them could be a common feature of these BVMOs.

In summary, we selected the novel BVMO_Lepto_ as a representative enzyme to explore the subclade shown in green in the phylogenetic tree of the current BVMOs (Fig. 1a), which had never been experimentally approached before. Thus, this work represents the first report of a confirmed BVMO that belongs to that subclade. The recombinant BVMO_Lepto_ accepted 28 out of the 36 reported ketones in whole-cell biotransformations. Our results indicate that BVMO_Lepto_ catalyzes the oxidation of linear- and branched- small and medium-length ketones as well as alkyl levulinates with excellent regioselectivity towards the corresponding normal esters, representing a very attractive biocatalyst for these transformations. This trend is conserved for linear aromatic ketones, except for acetophenone that it is not a substrate. Even though the cyclic and fused ketones tested seem to be moderate substrates, BVMO_Lepto_ was selective towards cycloketones with substituents in *α*-position to the carbonyl group and against other substitution patterns. This selective behavior will be the object of future studies.

## Additional file

**Additional file 1: Table S1.** BVMO sequences used to infer the phylogenetic tree. **Figure S1.** Multiple sequence alignment of BVMO_Lepto_ and representative BVMOs belonging to different clades. **Figure S2.** Homology modeling of the BVMO from *L. biflexa.*

## Abbreviations

BVMO: Baeyer–Villiger monooxygenase
BVMO_Lepto_: Baeyer–Villiger monooxygenase from *Leptospira biflexa*
CDMO: cyclododecanone monooxygenase
CHMO: cyclohexanone monooxygenase
CPMO: cyclopentanone monooxygenase
DNA: deoxyribonucleic acid
*ee*: enantiomeric excess
FAD: flavin adenine dinucleotide
GC-MS: gas chromatography-mass spectrometry
HAPMO: 4-hydroxyacetophenone monooxygenase
IPTG: isopropyl β-d-1-thiogalactopyranoside
LB: Luria–Bertani
na: not applicable
NADPH: reduced nicotinamide adenine dinucleotide phosphate
nc: no conversion
nd: not determined
PAMO: phenylacetonone monooxygenase
OD: optical density
PCR: polymerase chain reaction
SDS-PAGE: sodium dodecyl sulfate–polyacrylamide gel electrophoresis
