# Supplementary material for: Cloning and characterization of the Type I Baeyer–Villiger monooxygenase from Leptospira biflexa
Source: AMB Express. 2017 Apr 27;7:87. doi: 10.1186/s13568-017-0390-5 (PMC5407406; doi:10.1186/s13568-017-0390-5)
Supplement: Supplementary file 1 — Additional file 1: Table S1. BVMO sequences used to infer the phylogenetic tree. Figure S1. Multiple sequence alignment of BVMOLepto and representative BVMOs belonging to different clades. Figure S2. Homology modeling of the BVMO from L. biflexa. [file 13568_2017_390_MOESM1_ESM.docx]

**Cloning and characterization of the Type I Baeyer-Villiger monooxygenase from *Leptospira biflexa***

Romina D. Ceccoli^1^, Dario A. Bianchi^2^, Michael J. Fink^3,4^, Marko D. Mihovilovic^3^, Daniela V. Rial^1^*

^1^Área Biología Molecular, Departamento de Ciencias Biológicas, Facultad de Ciencias Bioquímicas y Farmacéuticas, Universidad Nacional de Rosario; CONICET, Rosario, Argentina

^2^Instituto de Química Rosario (IQUIR, CONICET-UNR); Departamento de Química Orgánica, Facultad de Ciencias Bioquímicas y Farmacéuticas, Universidad Nacional de Rosario, Rosario, Argentina

^3^Institute of Applied Synthetic Chemistry, Vienna University of Technology, Vienna, Austria

^4^Current affiliation: Department of Chemistry and Chemical Biology, Harvard University, Cambridge, MA, USA

Journal name: AMB Express

* Correspondence: Dr. Daniela V. Rial, Área Biología Molecular, Departamento de Ciencias Biológicas, Facultad de Ciencias Bioquímicas y Farmacéuticas, Universidad Nacional de Rosario, Suipacha 531, Rosario, S2002LRK, Argentina; CONICET, Argentina. E-mail: rial@inv.rosario-conicet.gov.ar, drial@fbioyf.unr.edu.ar

**Table S1** BVMO sequences used to infer the phylogenetic tree

| **Enzyme** | **Accession number** | **Amino acids identity** |
| --- | --- | --- |
| BVMO *Leptospira biflexa* | ABZ97795 | - |
| CHMO *Acinetobacter* sp. NCIMB 9871 | BAA86293 | 140/464 (30%) |
| BVMO *Acinetobacter radioresistens* S13 | ADF32068 | 117/435 (27%) |
| CHMO *Arthrobacter* sp. L661 | ABQ10653 | 136/472 (29%) |
| CHMO *Arthrobacter* sp. BP2 | AAN37479 | 139/472 (29%) |
| CHMO *Brachymonas petroleovorans* | AAR99068 | 142/488 (29%) |
| CHMO1 *Brevibacterium* sp. HCU | AAG01289 | 133/464 (29%) |
| CHMO2 *Brevibacterium* sp. HCU | AAG01290 | 123/499 (25%) |
| CPMO *Comamonas* sp. NCIMB 9872 | BAC22652 | 125/468 (27%) |
| BVMO3 *Dietzia* sp. D5 | AHE80562 | 100/417 (24%) |
| BVMO4 *Dietzia* sp. D5 | AGY78320 | 128/489 (26%) |
| ACMO *Gordonia* sp. TY-5 | BAF43791 | 130/494 (26%) |
| BVMO1 *Mycobacterium tuberculosis* H37Rv | CAA97398 | 201/461 (44%) |
| BVMO2 *Mycobacterium tuberculosis* H37Rv | CAA17436 | 96/346 (28%) |
| BVMO3 *Mycobacterium tuberculosis* H37Rv | CAB06212 | 108/426 (25%) |
| BVMO4 *Mycobacterium tuberculosis* H37Rv | CAB02175 | 176/488 (36%) |
| BVMO5 *Mycobacterium tuberculosis* H37Rv | CAA16134 | 185/471 (39%) |
| BVMO6 *Mycobacterium tuberculosis* H37Rv | CAA16141 | 120/426 (28%) |
| BVMO *Oceanicola batsensis* HTCC2597 | A3U3H1 | 129/486 (27%) |
| BVMO *Parvibaculum lavamentivorans* NCIMB13966 | A7HU16 | 136/497 (27%) |
| CHMO *Polaromonas* sp. JS666 | YP_552312 | 146/492 (30%) |
| CPDMO *Pseudomonas* sp. HI-70 | BAE93346 | 130/450 (29%) |
| CHMO *Pseudomonas aeruginosa* PAO1 | AAG04927 | 169/495 (34%) |
| BVMO *Pseudomonas fluorescens* DSM50106 | AAC36351 | 172/481 (36%) |
| HAPMO *Pseudomonas fluorescens* ACB | AAK54073 | 163/504 (32%) |
| HAPMO *Pseudomonas putida* JD1 | ACJ37423 | 157/477 (33%) |
| BVMO *Pseudomonas putida* KT2440 | AAN68413 | 113/420 (27%) |
| OTEMO *Pseudomonas putida* | AEZ35248 | 146/504 (29%) |
| BVMO *Pseudomonas veronii* MEK700 | ABI15711 | 117/442 (26%) |
| CHMO *Rhodococcus* sp. HI-31 | BAH56677 | 142/474 (30%) |
| CHMO *Rhodococcus* sp. TK6 | AAR27824 | 143/474 (30%) |
| CHMO *Rhodococcus* sp. Phi2 | AAN37491 | 141/474 (30%) |
| CHMO *Rhodococcus* sp. Phi1 | AAN37494 | 139/462 (30%) |
| BVMO1 *Rhodococcus jostii* RHA1 | ABG98452 | 128/495 (26%) |
| BVMO2 *Rhodococcus jostii* RHA1 | ABG96095 | 168/464 (36%) |
| BVMO3 *Rhodococcus jostii* RHA1 | ABG95050 | 135/506 (27%) |
| BVMO4 *Rhodococcus jostii* RHA1 | ABG94866 | 120/468 (26%) |
| BVMO5 *Rhodococcus jostii* RHA1 | ABG93916 | 162/481 (34%) |
| BVMO6 *Rhodococcus jostii* RHA1 | ABG93685 | 164/484 (34%) |
| BVMO7 *Rhodococcus jostii* RHA1 | ABG97785 | 183/488 (38%) |
| BVMO9 *Rhodococcus jostii* RHA1 | ABH00079 | 139/491 (28%) |
| BVMO10 *Rhodococcus jostii* RHA1 | ABH00083 | 148/504 (29%) |
| BVMO11 *Rhodococcus jostii* RHA1 | ABG98471 | 128/477 (27%) |
| BVMO12 *Rhodococcus jostii* RHA1 | ABG98876 | 167/457 (37%) |
| BVMO13 *Rhodococcus jostii* RHA1 | ABG95573 | 102/374 (27%) |
| BVMO14 *Rhodococcus jostii* RHA1 | ABG95240 | 121/464 (26%) |
| BVMO15 *Rhodococcus jostii* RHA1 | ABG94297 | 130/488 (27%) |
| BVMO16 *Rhodococcus jostii* RHA1 | ABG94724 | 114/430 (27%) |
| BVMO17 *Rhodococcus jostii* RHA1 | ABG97009 | 191/491 (39%) |
| BVMO18 *Rhodococcus jostii* RHA1 | ABG97176 | 150/499 (30%) |
| BVMO19 *Rhodococcus jostii* RHA1 | ABG97302 | 166/483 (34%) |
| BVMO20 *Rhodococcus jostii* RHA1 | ABG99184 | 123/492 (25%) |
| BVMO21 *Rhodococcus jostii* RHA1 | ABH00380 | 130/488 (27%) |
| BVMO24 *Rhodococcus jostii* RHA1 | ABG97104 | 145/489 (30%) |
| STMO *Rhodococcus rhodochrous* IFO 3338 | BAA24454 | 146/497 (29%) |
| CDMO *Rhodococcus ruber* SC1 | AAL14233 | 136/487 (28%) |
| BVMO PntE *Streptomyces arenae* | ADO85575 | 132/514 (26%) |
| BVMO PtlE *Streptomyces avermitilis* MA-4680 | BAC70705 | 122/447 (27%) |
| BVMO1 *Streptomyces coelicolor* A3(2) | CAB55657 | 129/416 (31%) |
| BVMO2 *Streptomyces coelicolor* A3(2) | CAB59668 | 178/485 (37%) |
| BVMO PenE *Streptomyces exfoliatus* | ADO85591 | 125/513 (24%) |
| BVMO (InfQ) *Streptomyces* sp. RI-77 | BAU98044.1 | 161/501 (32%) |
| PAMO *Thermobifida fusca* | Q47PU3 | 144/527 (27%) |
| CHMO *Xanthobacter* sp. ZL5 | CAD10801 | 129/442 (29%) |
| BVMO *Aspergillus clavatus* NRRL1 | XP_001270542 | 149/527 (28%) |
| BVMO210 *Aspergillus flavus* NRRL3357 | XP_002375343 | 123/466 (26%) |
| BVMO456 *Aspergillus flavus* NRRL3357 | XP_002375466 | 115/490 (23%) |
| BVMO619 *Aspergillus flavus* NRRL3357 | XP_002383043 | 133/492 (27%) |
| BVMO838 *Aspergillus flavus* NRRL3357 | XP_002375657 | 130/512 (25%) |
| BVMO1 *Aspergillus fumigatus* Af293 | XP_747160 | 136/483 (28%) |
| BVMO2 *Aspergillus fumigatus* Af293 | XP_746949 | 98/397 (25%) |
| BVMO3 *Aspergillus fumigatus* Af293 | XP_755274 | 135/489 (28%) |
| BVMO *Cyanidioschyzon merolae* 10D | BAM80902 | 148/543 (27%) |
| CAMO *Cylindrocarpon radicicola* ATCC 11011 | AET80001 | 138/483 (29%) |
| BVMO *Physcomitrella patens* | XP_001758613 | 154/532 (29%) |

PAMO MAG---------------------------------------------------------

CPMO MTT---------------------------------------------------------

CHMO M-----------------------------------------------------------

CDMO MTTS--------------------------------------------------------

HAPMO MSAFNTTLPSLDYDDDTLREHLQGADIPTLLLTVAHLTGDLQILKPNWKPSIAMGVARSG

BVMOLepto MTT---------------------------------------------------------

*

PAMO --------------------------------------------------QTTVDSRR--

CPMO --------------------------------------------------MTTMTTEQ--

CHMO ------------------------------------------------------------

CDMO ID-------REALRRKYAEERDKRI----RPDGNDQYIRLDHVDGWSHDPYMPITPRE--

HAPMO MDLETEAQVREFCLQRLIDFRDSGQPAPGRPTSDQLHILGTWLMGPVIEPYLPLIAEEAV

BVMOLepto ------------------------------------------------------------

PAMO ----------------QPPEEVDVLVVGAGFSGLYALYRLR-ELG-RSVHVIETAGDVGG

CPMO ----------LGMNN-SVNDKLDVLLIGAGFTGLYQLYHLR-KLG-YKVHLVDAGADIGG

CHMO ----------------SQKMDFDAIVIGGGFGGLYAVKKLRDELE-LKVQAFDKATDVAG

CDMO ----------------PKLDHVTFAFIGGGFSGLVTAARLR-ESGVESVRIIDKAGDFGG

HAPMO TAEEDLRAPRWHKDHVASGRDFKVVIIGAGESGMIAALRFK-QAG-VPFVIYEKGNDVGG

BVMOLepto ----------------SGFRNPSVVVIGAGMTGILLAIELE-KAGITDITILEKKHDLGG

. .:*.* *: .:. : . : *..*

PAMO VWYWNRYPGARCDIESIEYCYSFSEEVLQEWNWTERYASQPEILRYINFVADKFDLRSGI

CPMO IWHWNCYPGARVDTHCQIYQYSI-PELWQEFNWKELFPNWAQMREYFHFADKKLDLSKDI

CHMO TWYWNRYPGALTDTETHLYCYSWDKELLQSLEIKKKYVQGPDVRKYLQQVAEKHDLKKSY

CDMO VWYWNRYPGAMCDTAAMVYMPLLEETG---YMPTEKYAHGPEILEHCQRIGKHYDLYDDA

HAPMO TWRENTYPGCRVDINSFWYSFSFARGI-----WDDCFAPAPQVFAYMQAVAREHGLYEHI

BVMOLepto TWRENTYPGVACDIPAHMYTYSFAPNP----EWSHRFAHGDEIQAYFKRVSDEYKVTPKI

* * *** * * . : :: : : . :

PAMO TFHTTVTAAAFDEATNTWTV---DTNHGDRIRARYLIMASGQLSVPQLPNFPGLKDFAGN

CPMO SFNTRVQSAVFDEGTREWTV---RSIGHQPIQARFVIANLGFGASPSTPNVDGIETFKGQ

CHMO QFNTAVQSAHYNEADALWEV---TTEYGDKYTARFLITALGLLSAPNLPNIKGINQFKGE

CDMO LFHTEVTDLVWQEHDQRWRI---STNRGDHFTAQFVGMGTGPLHVAQLPGIPGIESFRGK

HAPMO RFNTEVSDAHWDESTQRWQLLYRDSEGQTQVDSNVVVFAVGQLNRPMIPAIPGIETFKGP

BVMOLepto HFNEAVTESSYQNA--KWTI---KTSKDKTYVSDFLISATGILHHPAKPNIPGLERFQGK

*: * ::: * : : : : * . * . *:: * *

PAMO LYHTGNWPHEPV----------DFSGQRVGVIGTGSSGIQVSPQIAKQAAELFVFQRTPH

CPMO WYHTALWPQEGV----------NMAGKRVAIIGTGSSGVQVAQEAALDAKQVTVYQRTPN

CHMO LHHTSRWPDD-V----------SFEGKRVGVIGTGSTGVQVITAVAPLAKHLTVFQRSAQ

CDMO SFHTSRWDYDYTGGDALGAPMDKLADKRVAVIGTGATAVQCVPELAKYCRELYVVQRTPS

HAPMO MFHSAQWDHD-V----------DWSGKRVGVIGTGASATQFIPQLAQTAAELKVFARTTN

BVMOLepto CFHTAEWDHS-V----------PLEGKRIGVIGTGSTAAQVIPEVMKLGKKVSVFQRTPQ

.*:. * . . .:*:.:****::. * .: * *:.

PAMO FAVPARNAPLDPEFLADLK----------KRYAEFREESRNTPGGTHRYQGPKSALEVSD

CPMO LALPMHQKQLSAEDNLRMK----------PELPAAFERRGKCFAGFDFDFIAKNATELSA

CHMO YSVPIGNDPLSEEDVKKIK----------DNYDKIWDGVWNSALAFGLNESTVPAMSVSA

CDMO AVDERGNHPIDEKWFAQIA-TPGWQKRWLDSFTAIWDGVLTDP----------SELAIEH

HAPMO WLLPTPDLHEKISDSCKWLLAH------VPHYSLWY------------------RVAMAM

BVMOLepto WIVKVPDTTYTEEDKKKWRKEPNL----LKRFHKWY------------------TFAVEQ

: . :

PAMO EELVETLERYWQEGG--PDILAAYRDILRDR-----------DANERVAEFIRNKIRNTV

CPMO AERTEILEELWNAGG-FRYWLANFQDYLFDD-----------KANDYVYEFWRDKVRARI

CHMO EERKAVFEKAWQTGGGFRFMFETFGDIATNM-----------EANIEAQNFIKGKIAEIV

CDMO ED---LVQDGWTALG--QRMRAAVGSVPIEQYSPENVQRALEEADDEQMERIRARVDEIV

HAPMO PQSVGFLEDVMVDVGYPPTELAVS------------------ARNDRLRQDISAWMEPQF

BVMOLepto TFSKAVIGKK------IPHMLMSF--------------------------LCKRNLRTSV

. : .

PAMO RD-PEVAERLVP--KGYPFGTKRLILEI-DYYEMFNRDNVHLVDTLSAPIETITPRGVRT

CPMO KD-PKVAEKLAPMKKPHPYGAKRPSLEQ-WYYEIFNQNNVTLVDVNETPVLRITEKGIVT

CHMO KD-PAIAQKLMP----QDLYAKRPLCDS-GYYNTFNRDNVRLEDVKANPIVEITENGVKL

CDMO TD-PATAAQLKA---WFRQMCKRPCFHD-DYLPAFNRPNTHLVDTGGKGVERITENGVVV

HAPMO ADRPDLREVLIP---DSPVGGKRIVRDNGTWISTLKRDNVSMI---RQPIEVITPKGICC

BVMOLepto KD-PELRKKLTP---NYRVGCKRVIVNS-TFYDAIQKPNADLV---TEGIEKITEKGVVT

* * * . ** . : ::: *. : : ** .*:

PAMO SEREY-ELDSLVLATGFDALTGALF---KIDIRGVGNVALKEKWA-AGPRTYLGLSTAGF

CPMO AEGEA-EFDLIVFATGFDAVTGGLT---SIDFRNNQGQSFKDVWS-DGIRTQLGVATAGF

CHMO ENGDFVELDMLICATGFDAVDGNYV---RMDIQGKNGLAMKDYWK-EGPSSYMGVTVNNY

CDMO AGVEY-EVDCIVYASGFEFLGTGYTDRAGFDPTGRDGVKLSEHWA-QGTRTLHGMHTYGF

HAPMO VDGTEHEFDLIVYGTGFHAS-KFLM---PINVTGRDGVALHDVWKGDDARAYLGMTVPQF

BVMOLepto KDGKLHELDVLVLATGFHPF-HFMR---PMNLTGENGISIETAWK-KKVQAYRSLFIPHF

*.* :: .:**. :: . . : * : .: :

PAMO PNLFFIAGPGSPSALSN-MLVSIEQHVEWVTDHIAYMFKNGLTRSEAVLEKEDEWVEHVN

CPMO PNLLFGYGPQSPAGFCN-GPSSAEYQGDLLIQLMNYLRDNNISRIEAQSEAQEEWSKLIA

CHMO PNMFMVLGPNGP--FTN-LPPSIESQVEWISDTIQYTVENNVESIEATKEAEEQWTQTCA

CDMO PNLFVLQLMQGAALGSN-IPHNFVEAARVVAAIVDHVLSTGTSSVETTKEAEQAWVQLLL

HAPMO PNMFCMYGPNTGLVVYSTVIQFSEMTASYIVDAVRLLLEGGHQSMEVKTPVFESYNQRVD

BVMOLepto PNFVLMLGPNTPIGNFS-VIAMSEVQTKYVLKIIEDWRRGKFNSIQATEEALHRFAAYLK

**:. . : : :. . :

PAMO E--IADETLYPMTASWYTGAN-VPG--KPRVFMLYVGGFHRYRQICDE-VAAKGYEGFVL

CPMO D--FWDSSLFPRAKSWYQGSN-IPG--KKVESLNFPLGLPTYISKFNE-SAEKGYAGFSL

CHMO N--IAEMTLFPKAQSWIFGAN-IPG--KKNTVYFYLGGLKEYRSALAN-CKNHAYEGFDI

CDMO D--HGRPLGNPECTPGYYNNEGKPAELKDRLNVGYPAGSAAFFRMMDHWLAAGSFDGLTF

HAPMO EGNALRAWGFSKVNSWYKNS-------KGRVTQNFPFTAVEFWQRTHS-VEPTDYQ---L

BVMOLepto KG-MAGTVWLGGCQSWYLDPDGDPAM--------WPY---TWKQWEKE-MKTPDYNDFSL

. . . : : : :

PAMO ------------T

CPMO A-----------S

CHMO QLQRSDIKQPANA

CDMO ------------R

HAPMO ------------G

BVMOLepto TT----------Q

**Figure S1** Multiple sequence alignment of BVMO_Lepto_ and representative BVMOs belonging to different clades. The alignment of PAMO from *T. fusca* (Q47PU3), CHMO from *Acinetobacter* sp. NCIMB 9871 (BAA86293), HAPMO from *P. fluorescens* ACB (AAK54073), CPMO from *Comamonas* sp. NCIMB 9872 (BAC22652), CDMO from *R. ruber* SC1 (AAL14233) and BVMO from *L. biflexa* (ABZ97795) is shown. The two Rossmann-fold motifs (GxGxxG/A) and the two consensus sequences of Type I BVMOs (G/AGxWxxxxF/YPG/MxxxD and FxGxxxHxxxWP/D) are highlighted in green and yellow, respectively. Protein sequences of BVMOs were aligned with MAFFT version 7 (Katoh and Standley 2013)


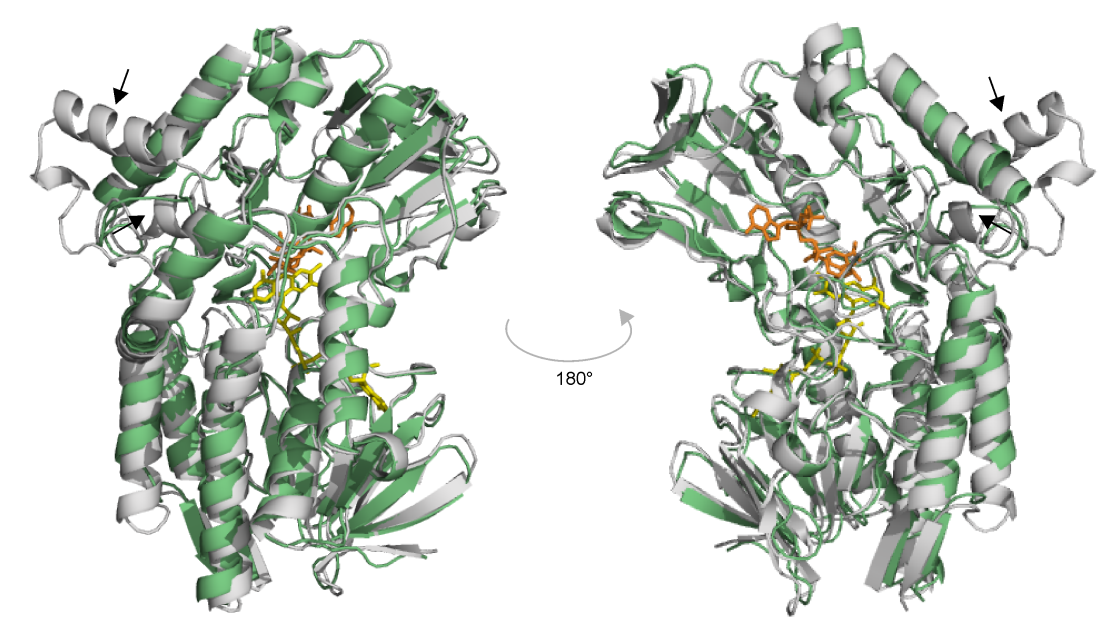


# Figure S2 Homology modeling of the BVMO from *L. biflexa.* Superimposition of the predicted three-dimensional structure of the BVMO_Lepto_ (green) and the reference CHMO from *Rhodococcus* sp. HI-31 (PDB 4rg3, gray) viewed from two sides. Arrows indicate the two helices absent in BVMO_Lepto_. The structural model of BVMO_Lepto_ was prepared by using Swiss-Model server (Biasini et al. 2014) and the quality of the model was tested using ProQ (Wallner et al. 2003)

References

Biasini M, Bienert S, Waterhouse A, Arnold K, Studer G, Schmidt T, Kiefer F, Cassarino TG, Bertoni M, Bordoli L, Schwede T (2014) SWISS-MODEL: modelling protein tertiary and quaternary structure using evolutionary information. Nucleic Acids Res 42:W252-W258

Katoh K, Standley DM (2013) MAFFT multiple sequence alignment software version 7: improvements in performance and usability. Mol Biol Evol 30:772-780

Wallner B, Fang H, Elofsson A (2003) Automatic consensus-based fold recognition using Pcons, ProQ, and Pmodeller. Proteins 53:534-541
